# Relationship between faculty characteristics and their entrepreneurial orientation in higher education institutions in Kuwait

**DOI:** 10.1186/s13731-022-00206-7

**Published:** 2022-02-04

**Authors:** Oualid Abidi, Khalil Nimer, Ahmed Bani-Mustafa, Sam Toglaw

**Affiliations:** 1 Department of Management, School of Business, Australian College of Kuwait, P.O. Box 1411, 13015 Safat, Kuwait; 2 Accounting and MIS Department, Gulf University for Science and Technology, 32093 Hawally, Kuwait; 3 Department of Mathematics and Physics, College of Engineering, Australian College of Kuwait, P.O. Box 1411, 13015 Safat, Kuwait; 4 Department of Marketing, School of Business, Australian College of Kuwait, P.O. Box 1411, 13015 Safat, Kuwait

**Keywords:** Entrepreneurial orientation, Higher education institutions, Experience, Gender, Qualifications, Scientific productivity

## Abstract

Considering intrapreneurship theory, this study aims to examine the extent to which the entrepreneurial orientation of faculty employed at Kuwaiti higher education institutions differ across their individual-level attributes. Faculty entrepreneurial orientation will be assessed at three levels, i.e., innovativeness, risk-taking, and proactivity. For this purpose, we surveyed a sample of 291 faculty from Kuwaiti colleges and universities. The core constructs were operationalized using scales validated in previous studies. The hypothesized relationships were tested using the structural equation modeling method. Our findings indicate that while female faculty are more proactive than men, males are innovative and risk-takers to some extent. Moreover, Ph.D. holders are more proactive and innovative than Master’s degree holders. The relationship between specialization and both innovativeness and risk-taking is significant only for business, but not for engineering. Teaching experience is more positively correlated with faculty proactivity. The number of scientific publications is negatively associated with faculty risk-taking propensity. Additionally, faculty who cumulated significant industry experience are proactive in identifying long-term opportunities and threats for their institutions. Having earned professional certifications is positively related to some aspects of innovativeness and proactivity. Finally, faculty who received their latest degree from a non-accredited institution are more active in realizing ideas at work.

## Introduction

The higher education sector is facing growing challenges and competition worldwide. In response, universities and colleges are intensifying their entrepreneurial activities with the input of their academic personnel to maintain sustainability and differentiation (Felgueira & Rodrigues, 2020; Ostojic & Leko Simic, 2021).

Kuwait provides an interesting case of development and growth in the higher education sector. According to the Private Universities Council in Kuwait (2021), the number of private universities and colleges has increased consistently in the last two decades. Despite the challenges imposed by COVID-19 pandemic, new universities where licensed in Kuwait and will start operation in a few years. Consequently, this has created a competitive environment and imposed a constant pressure on incumbent universities and colleges to maintain their market share, protect their positions in the market and follow more aggressive marketing and proactive approach to increase academic offering and recruit enough students.

In this regard, numerous Higher Education Institutions (HEI) undertook proactive working practices and embraced entrepreneurial philosophies to seize opportunities and reinforce their competitive position in the market (Chatterton & Goddard, 2000).

In fact, HEI are opting for a competence-centered approach that builds upon learning and knowledge transfer of their staff (Bratianu et al., 2020). Hence, faculty assume a significant role in this approach as they undertake value-adding activities with management support (Heinonen & Toivonen, 2007; Kearney & Meynhardt, 2016).

Scholarship conceptualized this role as employee *intrapreneurship* and defined it as an ‘agentic and strategic work behavior’ resulting in business venturing and strategic renewal (Gawke et al., 2019). For several researchers, employees’ intrapreneurship is underpinned by their entrepreneurial orientation (De Jong et al., 2015; Gawke et al., 2019; Kamil & Nasurdin, 2016; Meilani & Ginting, 2018; Mustafa et al., 2018; Neessen et al., 2019). The latter is defined as “*the extent to which individual workers proactively engage in the creation, introduction, and application of opportunities at work, marked by taking business-related risks*” (De Jong et al., 2015, p. 2).

Intrapreneurial employees develop new solutions opportunistically and execute them through small incremental changes (Heinze & Weber, 2016). In this sense, employees' entrepreneurial orientation in terms of innovation, proactiveness, risk-taking, and networking increases their organizations’ innovative performance (Almasri and Ahmad, 2020). As for HEI, faculty with entrepreneurial orientation can play an essential role in helping their institutions develop new academic programs that have potential market demand, interact with the industry and come up with innovative ideas and opportunities for growth and development. In addition, they can help their institutions achieve academic accreditation that enhances their credible position in the market and attract new students.

This study aims to probe into the relationships between individual characteristics and faculty entrepreneurial orientation (FEO). Despite the lack of research in this area, we intend to explore the faculty attributes that may have a stronger correlation with individual intrapreneurship in HEI in Kuwait. In accordance with previous studies, FEO will be assessed through three dimensions, i.e., innovativeness, proactivity, and risk-taking (De Jong et al., 2015; Farrukh et al., 2017; Gawke et al., 2019; Matloob & Raju, 2019; Meilani & Ginting, 2018). The existing body of research reveals that there is yet to be explored to understand intrapreneurship in academic context. Thus, this research addresses the question of how the faculty individual characteristics are related to their entrepreneurial orientation in HEI. It focuses on selected individual dimensions pertaining to gender, teaching experience, industry experience, scientific research productivity, professional certifications received, specialization (i.e., business or engineering), and accreditation standing of the school from which the highest degree was obtained.

The contributions of the present research are a depiction of a gap in the existing intrapreneurship literature for different reasons. First, it is one of the earliest studies to investigate the concept of entrepreneurial orientation among academics. To date, previous research predominately focused on professionals and employees working at manufacturing and other service-based firms. In addition, the largest span of studies conducted on intrapreneurship in academia focused on university spin-offs initiated by faculty members aiming at commercializing their scientific output through business venturing (e.g., Bezanilla et al., 2020; Kawamorita et al., 2020; Migliori et al., 2019). Second, this study is the first attempt to study faculty entrepreneurial orientation in the Gulf region. Third, the outcomes of this study will have practical utility as they may help HEI policy-makers identify individual characteristics associated with faculty intrapreneurship. For example, this would allow Kuwaiti HEI to refine their selection standards when recruiting new academic staff.

This paper is organized as follows. A literature review is presented in the first section. Then, the conceptual model is outlined with the research methodology. The findings of the study are described and analyzed then followed by a discussion. The paper concludes with remarks with the theoretical and practical implications as well as the future avenues of research.

## Literature review and hypotheses

Entrepreneurially oriented organizations depend on the contributions of proactive, risk-taking, and innovative employees who are likely to transform market opportunities into profitable solutions for their organizations (Neessen et al., 2019). These are described as intrapreneurial employees who produce new ideas at the right time and present them in the best shape (Shane & Venkataraman, 2000).

The emphasis on individual intrapreneurship concerns HEI, given that academics play a significant role in fulfilling the entrepreneurial orientation of their institutions through their participation in the dedicated councils and their exchange with the administrators (Clark, 2004). Faculty act as mentors for their students and guide towards developing projects with creative and useful outcomes for the industry and the community (Davis & Jacobsen, 2014).

Indeed, the challenging demands in public and private higher education sectors intensified the need for a more value-adding workforce. Therefore, the academic staff is expected to adopt an entrepreneurial orientation driven by innovation, proactiveness, and risk-taking (Hayat & Riaz, 2011). Faculty who embrace competitive mindsets and utilize cutting-edge teaching technologies are likely to increase the impact of their academic institutions in the business community (Meilani & Ginting, 2018). The use of e-assessments and social media technology are illustrations of rewarding digital tools implemented in HEI (Pillai and Prakash, 2017; Sharma & Pillai, 2017).

A large stream of literature studied the determinants of employee entrepreneurial orientation. Organizational variables were advocated such as management support (Mustafa et al., 2018; Sebora & Theerapatvong, 2010; ul Haq et al., 2018), job design (Mustafa et al., 2018), job autonomy (De Jong et al., 2015), proactive and pioneering leadership, decentralized and organic organizational structure (Nielsen et al., 2019), procedural justice and distributive justice (ul Haq et al., 2018). A more comprehensive study grouped those determinants into three categories: personal characteristics, job characteristics, and contextual characteristics (Kamil & Nasurdin, 2016). ALmasri and Ahmad (2020) included employee characteristics as moderating variables in their study of the impact of intrapreneurial behaviors on the innovative performance of commercial banks in Jordan. Those variables revolve around responsibility, effective communication, alertness, and awareness. Networking is another characteristic that is typical of intrapreneurial employees (Razavi & Ab Aziz, 2017). Also, the past intrapreneurial experience of an employee exerts a positive influence on his/her entrepreneurial orientation. Intrapreneurial experience is built up throughout learning processes conducted by employees within organizational realms to gain specific knowledge and develop leadership skills (Guerrero & Pena-Legazkue, 2013).

Little attention was given to the analysis of entrepreneurial orientation variability across individual characteristics in higher education. Previous studies pinpointed the role of (i) contextual factors revolving around top management support, work autonomy, flexibility, and reward system (Lizote et al., 2014) and (ii) organizational commitment, particularly affective commitment and normative commitment (Farrukh et al., 2017). Feola et. al. (2019) investigated the determinants of academic entrepreneurial intentions among Italian university researchers and found that psychological dimensions positively influence them. Building also on the theory of planned behavior, Miranda et. al. (2017) demonstrated that attitude towards entrepreneurship is the sole direct predictor of academic entrepreneurial intentions among Spanish university academics. This predictor is, in turn, influenced by the perceived utility, business experience, and creativity.

Based on their entrepreneurial characteristics, Rodrigues et. al. (2019) distinguished between five different groups of academics in HEI: downers, achievers, followers, defenders, and rebels. The most entrepreneurially engaged group of academics is the ‘achievers’ who significantly distinguished through their collaboration with external stakeholders and their capacity to mobilize resources for their research.

The study conducted by Ball (2019) suggests the positive impact of the proactiveness of public chief school business officials on the pro-business activity of their organizations (Ball, 2019).

Another research (Riggs, 2019) categorized academic staff into five groups of innovation adoption. It linked them with the demographic and academic characteristics (e.g., age, gender, race/ethnicity, teaching experience, and academic rank). Riggs (2019) found a positive correlation between faculty member innovativeness and being in the category of early majority adopters. However, only few studies focused on the effects of the academic profile of faculty on the degree of their entrepreneurial behavior (Ball, 2019; Hayter, 2013; Migliori et al., 2019; Rodrigues et al., 2019). Given the lack of evidence on the subject at hand, the present research will test a total of eight hypotheses that are discussed hereunder.

The first individual faculty characteristic that will be explored is gender. Gender was identified as a determinant of the faculty member’s attitude towards online education and computers. In their survey among pre-service classroom teachers enrolled in the ‘*Classroom Teacher Education*’ program at a local university in Turkey, Yorulmaz et. al. (2016) found that the state of their innovativeness varies according to gender. Aldahdouh et. al. (2020) demonstrated that males tend to be earlier adopters of new technological devices among university members in Finland. In the same vein, Sánchez and Licciardello (2012) found that men showed greater levels of entrepreneurial self-efficacy than women. They felt more oriented to create a new venture than women. Other studies, such as Cromie (1987), did not find notable differences between males and females in dimensions of the entrepreneurial process, such as the need for achievement, locus of control, and planning. Therefore, we could refer to the existence of mixed results in previous studies about the effect of faculty gender on their entrepreneurial behaviors.

### **Hypothesis 1**

There is a relationship between gender and the three dimensions of faculty entrepreneurial orientation (innovativeness, risk-taking, and proactivity).

In accordance with their need to have a certain degree of division of labor, organizations may rely on people with different educational backgrounds. The educational accomplishment of staff members is likely to define the extent of their knowledge, skills and abilities. Nonetheless, evidence shows that employee creativity and willingness to innovate is not necessarily correlated with possessing higher educational qualifications (Muange & Kiptoo, 2020). For instance, Boadi and Osarfo (2019) demonstrated that holding a first degree helps in increasing the financial performance of the banks. Interestingly, they found also that an increasing number of board members with Doctor of Philosophy (Ph.D.) is associated with adverse effects on banks’ financial performance. Investigating the type of influence of academic qualifications on organizational members entrepreneurial orientation in HEI settings would add to the existing body of knowledge. Thus, Hypothesis 2 is formulated.

### **Hypothesis 2**

There is a relationship between the educational qualifications and the three dimensions of faculty entrepreneurial orientation (i.e., innovativeness, risk-taking, and proactivity).

In regard to the faculty specialization (i.e., business or engineering), the present study will help determining which one has the strongest association with the three dimensions of faculty entrepreneurial orientation. On one hand, engineering faculty could be assumed to be more entrepreneurially oriented since they essentially deal with applied knowledge with better funding perspective from either governmental bodies or private firms. For instance, they may involve their students in developing solutions and drafting new technologies for existing needs. This aligns with the approach of Education 4.0 applied in engineering education based on four components, i.e., competencies, learning methods, infrastructure, and information and communication technologies (Miranda et al., 2021). For instance, software engineering education is adjusting to the industry needs by using project-based learning and by integrating software engineering trends such as global software engineering, and lean software startup (Cico et al., 2021). Business faculty, on the other hand, may be expected to have solid business acumen and knowledge of macro-level environmental factors. This could enable them to use practical entrepreneurial education approaches to guide their students in business venturing. Evidence shows indeed that adapted entrepreneurial education supports the entrepreneurial intentions among students (Maresch et al., 2016). Hence, the third hypothesis states the following.

### **Hypothesis 3**

There is a relationship between the specialization and the three dimensions of faculty entrepreneurial orientation (i.e., innovativeness, risk-taking, and proactivity).

In their analysis of the factors affecting the competence profile of university teachers, Fernández-Cruz and Rodríguez-Legendre (2021) found that faculty with more than 20 years of teaching experience show a stronger innovation competence profile. This is an evidence that cumulating experience in teaching allows faculty to constantly upgrade their pedagogy and figure out novel ways of educating their students. Nonetheless, the COVID-19 implied that all HEI worldwide shift towards remote teaching. In this vein, experience gained in conventional education settings might be less decisive in what education will look like in the post-pandemic phase. As a matter of fact, Adedoyin and Soykan (2020) believe that online learning is going to be sustainable after the pandemic, maybe through a hybrid approach that should be different from the emergency response-migration during the crisis. Hypothesis 4 is posed accordingly to determine the nature of teaching experience effect.

### **Hypothesis 4**

There is a relationship between the teaching experience and the three dimensions of faculty entrepreneurial orientation (i.e., innovativeness, risk-taking, and proactivity).

The following characteristic concerns the amount of scientific research produced by the faculty. The research record of teaching staff is assumed to leverage their intrapreneurial potential and contribute to the growth of their institutions. Researching about real organizational issues may allow faculty to incorporate their findings within their courses and to engage students in different aspects of their research endeavors. This postulate has support in the literature. Indeed, Rubin and Callaghan (2019) found an association between faculty’s entrepreneurial orientation and their research productivity, except for the innovativeness dimension. Bojko et. al. (2021) demonstrated that research productivity has a positive impact on academic entrepreneurship, though this effect is stronger among the ones who are engaged locally. Those who are eager to disseminate their research globally seem to have more difficulty to reconcile those aims with academic entrepreneurship. Through Hypothesis 5, the present study will determine the extent to which research productivity supports or limits the entrepreneurial orientation of faculty in Kuwaiti HEI.

### **Hypothesis 5**

There is a relationship between the research record and the three dimensions of faculty entrepreneurial orientation (i.e., innovativeness, risk-taking, and proactivity).

The next individual characteristic that will be investigated pertains to the faculty’s amount of professional experience. Hayter (2013) demonstrated that academics with significant experience in business consulting are more likely to succeed in transforming their knowledge into profitable product markets. Similarly, managerial experience is predicting the entrepreneurial intentions of scientists (Huszár et al., 2016). Migliori et. al. (2019) found that faculty’s market orientation positively affects the performance of the spin-offs created by Italian universities. Although the related scholarship is pointing towards a potential positive correlation between the degree of faculty’s involvement with the industry and his/her personal intrapreneurship, we may expect different effects in the Kuwaiti higher education sector as professional occupations among faculty are usually overridden by enormous teaching load demands in addition to institutional restrictions on exercising extra-academic activities. Thus, we formulate the following hypothesis.

### **Hypothesis 6**

There is a relationship between the industry experience and the three dimensions of faculty entrepreneurial orientation (i.e., innovativeness, risk-taking, and proactivity).

The next assumption goes in the same direction as the focus is on the role of professional certifications in enhancing the faculty’s inclination towards being innovative, risk-taker and proactive. Indeed, the race for international accreditations among universities and colleges worldwide increased the need for academics to be certified as professionals in their respective specializations. Those recognitions are also being promoted by their institutions to progress in international university ranks (Kamayanti, 2020). For instance, accounting faculty holding professional certifications consider that although professional certifications help them bridging the gap between theory and professional requirements in their teaching, it does nothing to support their research output (Bergner et al., 2020). Hence, our focus in Hypothesis 7 below will focus on determining the type of influence that professional certifications exert on academics in Kuwaiti HEI.

### **Hypothesis 7**

There is a relationship between the number of professional certifications and the three dimensions of faculty entrepreneurial orientation (i.e., innovativeness, risk-taking, and proactivity).

Last, faculty who graduated from internationally accredited institutions are thought to possess greater capacity to contribute to the growth of their HEI in Kuwait. They were supposedly exposed to advanced learning experiences that could be leveraged once employed. This is in accordance with Kafaji (2020) who recognizes that—from business students’ perspective—accreditations can benefit their performance, motivation, and career prospects. Other views contend, however, that academic accreditations can have counterproductive effects on teaching innovation and the quality of learning due to its subtle controlling and bureaucratic nature (Harvey, 2004). Hypothesis 8 is established accordingly to examine the type of effect of the accreditation status of last attended institution on faculty entrepreneurial orientation.

### **Hypothesis 8**

There is a relationship between the accreditation standing of the School attended for the latest academic degree and the three dimensions of faculty entrepreneurial orientation (i.e., innovativeness, risk-taking, and proactivity).

## Research methodology

### Research design

As the main aim of this study is to examine the impact of individual-level variables on the entrepreneurial orientation of faculty in higher education institutions in Kuwait, the research design relied on primary data collected using a survey to study the relationship between FEO and EOHEI.

### Sampling frame, instrument, and data

We have designed our questionnaire based on the aims of our study, a wide-ranging literature review, and similar contents of questionnaires used in previous studies (Farrukh et al., 2017; De Jong et al., 2015; Hughes and Morgan, 2007; Smeenk et al., 2006, 2008). The questionnaire includes two main sections; the first one includes questions related to Faculty Characteristics at the individual level. The second section was related to faculty entrepreneurial orientation, which includes three sub-sections; innovativeness, risk-taking, and proactivity. To measure the level of three proxies of faculty entrepreneurial orientations, faculty members were asked to indicate their level of agreeableness (on the 5-Likert scale, 1 = strongly disagree and 5 = strongly agree) to a certain number of statements in each section regarding these issues. The questionnaire survey was sent to 341 faculty members in Kuwait universities’ engineering and business faculties (private and public). Choosing the engineering and business’ faculties only was to ensure comparability and consistency as these two faculties have existed in most Kuwait universities. All of the survey questionnaires were sent to the formal email of faculties from June to October 2020. Each email includes a covering letter and the questionnaire link. We have received a total of 291 usable questionnaires by the end of October 2020, representing an 85 percent response rate. The sample size and response rate were found to be acceptable (Cochran, 1977). The characteristics of the respondents were as follows (Table 1).

**Table 1:** Distribution of the sample

Variable	Category			*n*	%
Gender	Male			196	67.4
	Female			95	32.6
Education level	Masters/MBA			64	26.8
	Ph.D.			186	73.2
Age	25–34 years			53	18.2
	35–44 years			152	52.2
	≥ 45			86	29.6
Employer	HEI 1			43	14.8
	HEI 2			42	14.4
	HEI 3			26	8.9
	HEI 4			28	9.6
	HEI 5			95	32.6
	HEI 6			34	11.7
	HEI 7			14	4.8
	HEI 8			9	3.1
Academic field	Business			210	72.2
	Engineering			81	27.8
Number of publications	0–1			120	41.2
	2–5			128	43.9
	6–10			26	8.8
	More than 10			17	5.9
Management style	Authority–compliance (efficiency)—1			81	27.8
	Impoverished management (laissez-faire management)—2			38	13.1
	Country club management (friendly atmosphere)—3			40	13.7
	Middle of the road management (balancing work and people)—4			79	27.1
	Team management (trust and respect)—5			53	18.2
Descriptive statistics					
Teaching experience	Mean = 12.6	Stdev = 6.2	Min = 0	Max = 33	
Industrial experience	Mean = 4.5	Stdev = 4.6	Min = 0	Max = 25	

The HEI type of ownership variable (private/public) was dropped since the responses received from private HEI staff members largely outnumbered the other category. Only 10 respondents from Kuwaiti public HEI completed the questionnaire

## Analysis and results

The collected data were cleaned and analyzed using SPSS 24.0 and AMOS 25 using Structural Equation Modeling (SEM) with maximum likelihood estimation to test the research model and our main hypothesis. SEM is used to have some advantages and proven to be a powerful technique over multiple regression modeling. First, SEM allows modeling multi-equation regression models and various measures of concepts that fit well with our research model. Second, the SEM model correlation among variables is used in the model.

### Exploratory factor analysis and scale reliability analysis

As the study is conducted in Kuwait, all items are reanalyzed and validated with internal reliability values, Cronbach Alpha, and Bartlett’s test (Hoque et al., 2018). The adopted methodology used in modeling the data follows Moussa et. al. (2019). Evaluating and validating our instrument for our constructs' recommended items, 291 respondents have been used in this study. The FEO construct, with a total of 15 items, is illustrated in Table 2.

**Table 2:** Theoretical background/support for scale items, variables, and constructs

Item description	Theoretical background
Construct/variable: faculty entrepreneurial orientation (FEO)	
Innovativeness—6 items	Farrukh et. al. (2017)—4 items De Jong et. al. (2015)—2 items
Risk-taking—6 items	Farrukh et. al. (2017)—3 items De Jong et. al. (2015)—3 items
Proactivity—3 items	De Jong et. al. (2015)

Sample adequacy for factor analysis is checked and found to be significant at a 5 percent level using Kaiser–Meyer–Olkin (KMO) and Bartlett’s sphericity with a Chi-square value of 1290.7 (df = 105). KMO statistic (0. 813) is greater than the cut of value (0.50) (Hair et al., 2013). The results obtained by running factor analysis using Varimax rotation (Costello and Osborne, 2005) resulting in cross-loadings (> 0.40) or communalities (< 0.30) (Hair et al., 2012) and eigenvalues more than one were taken as references. Three factors were extracted with a total explained variance of 64.7 percent.

Based on our instrument’s internal reliability and exploratory factor analysis, all the items were retained except 18 and 23 in the construct/variable FEO. Cronbach alpha values were also above the cut of the value of 0.7 for all variables and constructs with a value of 0.73.

### Measurement and theoretical model estimation and fit

This study assumed that FEO is a latent construct and cannot be directly measured; instead, it must be assessed using a set of explicit three variables (innovativeness, risk-taking, and proactivity). Those three variables are also evaluated by items, as shown in Table 2. Six items for each of innovativeness and risk-taking, and three items for proactivity. Besides the validity and reliability tests above using factor analysis and Cronbach alpha, the measurement model is also used to validate our theoretical model, consisting of our primary constructs used in the study (Table 2). Table 3 shows the results of the measurement model for our primary FEO construct. FEO construct indicators and variables were found highly significant at the alpha level of 0.001, as shown in Table 3. The goodness of fit (GOF) is satisfactory based on the goodness-of-fit criteria (GFI, AGFI, CFI, and NFI > 0.90 and RMSEA < 0.07), suggesting that the hypothesized measurement model fits the data very well.

**Table 3:** Statistics of measurement analysis (FEO)

Constructs, variables, and items	Regression weights	Standardized weights	The goodness-of-fit indicators		Acceptable standard fit
PRO à FEO	1.99**	1.00	GFI	0.969	> 0.90
RTAKING à FEO	1.67*	1.00	AGFI	0.900	> 0.90
INNOV à FEO	1.00	1.00	CFI	0.983	> 0.90
Innovativeness					
Inn12	1.000	0.298	NFI	0.952	> 0.90
Inn13	0.260	0.071	RMSEA	0.040	< 0.07
Inn14	− 0.41	− 0.090			
Inn15	1.04**	0.363			
Inn16	2.44***	0.552			
Inn17	2.18**	0.659			
Risk-taking					
RT20	0.16	0.08			
RT21	0.891***	0.304			
RT22	1.00***	0.354			
Proactivity					
Pro24	1.36***	0.667***			
Pro25	0.50***	0.295***			
Pro26	1.00***	0.588***			

*GFI* goodness-of-fit index, *AGFI* adjusted goodness-of-fit index, *CFI* comparative fit index, *NFI* normed fit index, *RMSEA* root mean square residual

*Significant at the 0.10 level

**Significant at the 0.05 level

***Significant at the 0.01 level

The results show that proactivity has the highest contributor to the FEO construct. This confirms Cleverley-Thompson’s (2016) findings, pinpointing proactivity as a primary entrepreneurial characteristic of academics. Moreover, *Inn17* and *Inn16* have the highest and significant contribution to innovativeness variable (respectively, “*I search out new techniques, technologies and/or product ideas*”; “*In the course of my work, I develop new processes, services or products*”), and the least contributing two items (and not significant) are *Inn13* and *Inn12* (respectively, “*I attempt to convince people to support an innovative idea*”; “*In the course of my work, I generate creative ideas*”). For Risk-taking, the highest (and significant) contribution comes from *RT22* (“*I take risks in my job*”) and the least contribution (and not significant) for *RT20* (“*In the course of my work, I will take calculated risks despite the possibility of failure*”). Finally, the highest and significant contribution comes from *Pro24* (“*I identify long-term opportunities and threats for the company*”), and the least and significant contribution comes from *Pro25* (“*I am known as a successful idea seller*”).

### Structural model results subject to faculty characteristics and hypothesis testing

After checking the theoretical measurement model, the data were analyzed using SEM to investigate the main research hypotheses. After the first step in evaluating our theoretical model and assessing our constructs (FEO) from the hypothesized variables and items, the structural model data are used to test if the relationships between our items and/variables are different subject to the respondent’s characteristics. A comparison of males and females using SEM for our primary construct (FEO) is summarized in Table 4, along with the GOF. GOF indicates that our hypothesized model fits the data well, and all GOF indicators are above the recommended values (Bagozzi and Yi, 2012).

For both males and females, their proactivity draws on their contributions in identifying long-term threats and opportunities for their institutions (*RT24*). However, our results show that female faculty are more engaged in this undertaking (*p*-value < 0.10).

About risk-taking, males are inclined to take calculated risks, although this could be conducive to failure (*RT20*) (*p*-value < 0.001). Nonetheless, the results indicate that for females, ‘taking calculated risks despite the possibility of failure’ is not a significant item in the risk-taking dimension of FEO.

Similarly, the innovativeness-related items of “*I convince people to support an innovative idea*” (i.e., *Inn13*) and “*I visualize a clear plan of action when I consider ways to make a new idea happen*” (i.e., *Inn14*) are only significant for male faculty (*p*-value < 0.05). Thus, Hypothesis 1 is partially supported (Table 4).

**Table 4:** SEM results for FEO construct subject to gender

	Estimate Male	Estimate Female	*z*-score	Goodness-of-fit indicators		Acceptable standard fit
F_Proa ← FacEntre	1.365***	1.994**	0.677	GFI	0.97	> 0.90
F_RTaking ← FacEntre	0.593***	1.670*	1.126	AGFI	0.90	> 0.90
Pro25 ← F_Proa	0.839***	0.495***	− 1.567	CFI	0.983	> 0.90
Pro24 ← F_Proa	0.702***	1.355***	1.866*	NFI	0.952	> 0.90
RT21 ← F_RTaking	1.118***	0.891***	− 0.513	RMSEA	0.04	< 0.07
RT20 ← F_RTaking	1.548***	0.159	− 2.711***			
Inn13 ← F_innova	1.338***	0.260	− 2.224**			
Inn14 ← F_innova	1.186***	− 0.406	− 2.27**			
Inn15 ← F_innova	1.021***	1.042**	0.040			
Inn16 ← F_innova	1.490***	2.439***	1.086			
Inn17 ← F_innova	1.112***	2.178**	1.067			

***p-value < 0.01

**p-value < 0.05

*p-value < 0.10

The analysis of the relationship between FEO and faculty qualifications is reported in Table 5. While the results show that proactivity is a significant component for both masters and Ph.D. holders of their intrapreneurial orientation, it is noteworthy to recognize that proactivity’s weight as an FEO component is significantly higher for Ph.D. holders than master holders a *p*-value < 0.05.

**Table 5:** SEM results for FEO construct subject to qualification

	Estimate Master	Estimate Ph.D.	*z*-score	Goodness-of-fit indicators		Acceptable standard fit
F_Proa ← FacEntre	0.905***	1.861***	2.223**	GFI	0.977	> 0.90
F_RTaking ← FacEntre	0.521***	0.894***	0.992	AGFI	0.904	> 0.90
Pro25 ← F_Proa	0.461***	0.801***	1.672*	CFI	0.995	> 0.90
Pro24 ← F_Proa	1.418***	0.649***	− 2.917***	NFI	0.966	> 0.90
RT21 ← F_RTaking	1.316***	1.026***	− 0.572	RMSEA	0.023	< 0.07
RT20 ← F_RTaking	0.879**	1.188***	0.573			
Inn13 ← F_innova	0.772***	1.456***	1.844*			
Inn14 ← F_innova	0.396*	1.371***	2.428**			
Inn15 ← F_innova	0.580***	1.305***	2.266**			
Inn16 ← F_innova	1.318***	1.874***	1.396			
Inn17 ← F_innova	0.676***	1.720***	2.701***			

***p-value < 0.01

**p-value < 0.05

*p-value < 0.10

The table above also reveals that proactivity for both master's and Ph.D. holders stems from their capacity to be “*a successful idea seller*” (*Pro25*). However, this item is significantly more relevant to Ph.D. holders’ proactivity than Master’s holders.

Identifying long-term opportunities and threats for the institution (*Pro24*) is an essential and significant aspect of proactivity for both degree holders (Masters and Ph.D.). Still, it is significantly more related to proactivity for master holders than Ph.D. holders.

For both Master and Ph.D. holders, innovativeness is significantly manifested through the following actions: attempting to convince people to support an innovative idea (*Inn13*); visualizing a clear plan of action when considering ways to make a new idea happen (*Inn14*); being particularly good at realizing ideas at work (*Inn15*); and searching out new techniques, technologies and/or product ideas (*Inn17*). Those aspects are significantly more relevant to innovativeness for Ph.D. holders than Masters. Overall, our results largely support Hypothesis 2 (Table 5).

The results presented in Table 6 focus on understanding the extent to which faculty affiliation is likely to affect FEO. This study provides minor support to Hypothesis 3 as they show slight differences, but not significant between business and engineering faculty. The most notable differences concern three risk-taking dimensions. First, business faculty “*regularly go for the big win even when the risks are considerably high*” if large interests are at stake (*RT21*). This dimension is insignificant for engineering faculty. Similarly, the results show that—contrary to engineering faculty (insignificant influence). Innovativeness among business faculty is effective through their attempts to convince people to support an innovative idea (*Inn13*) and their inclination to visualize a clear action plan when they strive to concretize a new idea (*Inn14*) (Table 6).

**Table 6:** SEM results for FEO construct subject to school

	Estimate Bus	Estimate Eng	*z*-score	Goodness-of-fit indicators		Acceptable standard fit
F_Proa ← FacEntre	1.553***	1.770**	0.273	GFI	0.972	> 0.90
F_RTaking ← FacEntre	0.842***	1.176*	0.461	AGFI	0.902	> 0.90
Pro25 ← F_Proa	0.621***	0.988***	1.233	CFI	0.961	> 0.90
Pro24 ← F_Proa	0.790***	0.809***	0.061	NFI	0.961	> 0.90
RT21 ← F_RTaking	0.986***	0.511	− 1.101	RMSEA	0.022	< 0.07
RT20 ← F_RTaking	0.843***	0.748**	− 0.223			
Inn13 ← F_innova	1.260***	0.571	− 1.452			
Inn14 ← F_innova	0.991***	0.607	− 0.611			
Inn15 ← F_innova	1.017***	0.623	− 0.893			
Inn16 ← F_innova	1.607***	1.485***	− 0.210			
Inn17 ← F_innova	1.262**	1.176**	− 0.171			

***p-value < 0.01

**p-value < 0.05

*p-value < 0.10

Table 7 presents the results pertaining to the association between FEO and faculty previous experience. Our results partially endorse the fourth hypothesis. They indicate few differences between experienced faculty (with more than ten years of teaching experience) and lower experience (less than ten years of experience). For more experienced academics, proactivity is significantly more contributive to their intrapreneurial orientation. In comparison to less experienced faculty, innovativeness among experienced faculty is significantly more driven by their attempts “*to convince people to support an innovative idea*” (*Inn13*) and their efforts in searching search out new techniques, technologies, and/or “*product ideas*” (*Inn17*).

**Table 7:** SEM results for FEO construct subject to experience

	Estimate Exp1	Estimate Exp2	*z*-score	Goodness-of-fit indicators		Acceptable standard fit
F_Proa ← FacEntre	0.831***	1.542***	2.324**	GFI	0.968	> 0.90
F_RTaking ← FacEntre	0.534***	1.010***	1.448	AGFI	0.901	> 0.90
Pro25 ← F_Proa	0.674***	0.907***	0.984	CFI	0.993	> 0.90
Pro24 ← F_Proa	0.636***	0.903***	1.223	NFI	0.953	> 0.90
RT21 ← F_RTaking	1.138***	0.654***	− 1.241	RMSEA	0.024	< 0.07
RT20 ← F_RTaking	0.799***	0.745***	− 0.155			
Inn13 ← F_innova	0.457***	1.436***	3.278***			
Inn14 ← F_innova	0.993***	0.598***	− 1.406			
Inn15 ← F_innova	0.883***	0.679***	− 0.854			
Inn16 ← F_innova	1.323***	1.610***	0.919			
Inn17 ← F_innova	0.635***	1.246***	2.359**			

***p-value < 0.01

**p-value < 0.05

*p-value < 0.10

As shown in Table 8, Hypothesis 5 is slightly confirmed. The only difference between faculty with a low number of publications and the more productive ones pertains to risk-taking, representing an essential aspect of intrapreneurial orientation for the former category but not for the latter. In the same way for risk-taking, it is significantly important for faculty with a low number of publications to take calculated risks in the course of their work despite the possibility of failure (*RT20*) and to go for the big win even when the risks are considerably high (*RT21*). Our findings show that those risk-taking dimensions are not crucial for faculty with a higher number of publications (Table 8).

**Table 8:** SEM results for FEO construct subject to publication

	Estimate Pub1	Estimate Pub2	*z*-score	Goodness-of-fit indicators		Acceptable standard fit
F_Proa ← FacEntre	1.511***	1.184***	− 0.685	GFI	0.990	> 0.90
F_RTaking ← FacEntre	1.571***	− 0.310	− 3.709***	AGFI	0.991	> 0.90
Pro25 ← F_Proa	0.854***	0.787**	− 0.174	CFI	0.999	> 0.90
Pro24 ← F_Proa	0.731***	0.862**	0.363	NFI	0.987	> 0.90
RT21 ← F_RTaking	0.885***	2.347	0.541	RMSEA	0.011	< 0.07
RT20 ← F_RTaking	0.589***	− 1.241	− 0.817			
Inn13 ← F_innova	1.269***	1.121*	− 0.220			
Inn14 ← F_innova	0.856***	1.131***	0.598			
Inn15 ← F_innova	1.275***	0.778*	− 1.021			
Inn16 ← F_innova	1.250***	1.388**	0.229			
Inn17 ← F_innova	1.183***	1.269**	0.151			

***p-value < 0.01

**p-value < 0.05

*p-value < 0.10

A summary of the relationship between FEO and faculty industry experience can be seen in Table 9. With respect to industry experience (i.e., Hypothesis 6), the main difference concerns proactivity among highly experienced faculty. The results show that this group of faculty is significantly engaging in identifying long-term opportunities and threats for their institutions (*Pro24*). This dimension is, however, insignificant for the faculty with low industry experience (Table 9).

**Table 9:** SEM results for FEO construct subject to industrial experience

	Estimate Ind_Exp1	Estimate Ind_Exp2	*z*-score	Goodness of fit indicators		Acceptable standard fit
F_Proa ← FacEntre	2.448**	0.991***	− 1.281	GFI	0.992	> 0.90
F_RTaking ← FacEntre	5.342*	0.546***	− 1.488	AGFI	0.929	> 0.90
Pro25 ← F_Proa	0.735***	0.966***	0.806	CFI	0.999	> 0.90
Pro24 ← F_Proa	− 1.012	1.081***	3.275***	NFI	0.989	> 0.90
RT21 ← F_RTaking	1.044***	0.574***	− 1.475	RMSEA	0.011	< 0.07
RT20 ← F_RTaking	1.102***	0.378	− 1.641			
Inn13 ← F_innova	1.856	0.897***	− 0.531			
Inn14 ← F_innova	2.641**	0.631***	− 1.631			
Inn15 ← F_innova	4.713*	0.685***	− 1.405			
Inn16 ← F_innova	1.668*	1.255***	− 0.467			
Inn17 ← F_innova	5.345*	0.663***	− 1.281			

***p-value < 0.01

**p-value < 0.05

*p-value < 0.10

Table 10 highlights the weak significance of the number of professional certificates earned by faculty on their entrepreneurial orientation. The results show little support for Hypothesis 7. There are no major differences between faculty with one or less professional certificate and those holding more than one professional certificate except for three dimensions. Concerning innovativeness, faculty with more than one professional certificate are significantly eager to convince people to support an innovative idea, while those with fewer certificates do not (*Inn13*). All faculty—irrespective of the number of professional certifications that they have—are active in searching out new techniques, technologies, and/or “*product ideas*” (*Inn17*). However, this innovativeness aspect is significantly more determinant for faculty with more than one certificate than those with fewer professional certificates. Both categories (≤ 1 and > 1 professional certificate) are successful idea sellers regarding proactivity dimensions. However, that feature is significantly stronger for faculty with more than one professional certificate than those belonging to the second category (≤ 1 certificate; Table 10).

**Table 10:** SEM results for FEO construct subject to certificates

	Estimate Cert1	Estimate Cert2	*z*-score	Goodness of fit indicators	Acceptable standard fit	
F_Proa ← FacEntre	1.167***	1.485***	0.749	GFI	0.990	> 0.90
F_RTaking ← FacEntre	0.981***	0.931***	− 0.113	AGFI	0.991	> 0.90
Pro25 ← F_Proa	0.464***	1.128***	2.618***	CFI	0.999	> 0.90
Pro24 ← F_Proa	0.588***	0.773***	0.754	NFI	0.987	> 0.90
RT21 ← F_RTaking	0.615***	0.737***	0.402	RMSEA	0.011	< 0.07
RT20 ← F_RTaking	0.509	1.104**	1.053			
Inn13 ← F_innova	0.190	1.867***	3.324***			
Inn14 ← F_innova	0.854***	0.732***	− 0.321			
Inn15 ← F_innova	0.777***	1.184***	1.007			
Inn16 ← F_innova	0.933***	1.643***	1.557			
Inn17 ← F_innova	0.556**	1.534***	2.206**			

***p-value < 0.01

**p-value < 0.05

*p-value < 0.10

Table 11 provides the results for the relationship between FEO and the accreditation status of the school from which the respondent completed his/her latest degree. The only significant fact about the latest School's accreditation status (AACSB/ABET-accredited versus non-accredited School) is related to innovativeness. Our findings demonstrate that regardless of the School's accreditation standing, all faculty perceive themselves as particularly good at realizing work ideas. Nonetheless, the results indicate that this capacity is more substantive among faculty who received their latest degree from a non-accredited institution than those who graduated from accredited schools. Therefore, Hypothesis 8 gained little support in our findings.

**Table 11:** SEM results for FEO construct subject to the accreditation status of the latest attented HEI

	Estimate Acc1	Estimate Acc2	*z*-score	Goodness-of-fit indicators		Acceptable standard fit
F_Proa ← FacEntre	1.765***	1.407***	− 0.710	GFI	0.965	> 0.90
F_RTaking ← FacEntre	1.524***	0.744***	− 1.405	AGFI	0.903	> 0.90
Pro25 ← F_Proa	0.565***	0.696***	0.624	CFI	0.987	> 0.90
Pro24 ← F_Proa	0.942***	0.646***	− 1.314	NFI	0.946	> 0.90
RT21 ← F_RTaking	0.837***	0.737***	− 0.373	RMSEA	0.031	< 0.07
RT20 ← F_RTaking	0.851***	0.681***	− 0.591			
Inn13 ← F_innova	1.058***	1.101***	0.127			
Inn14 ← F_innova	1.098***	0.767***	− 0.803			
Inn15 ← F_innova	1.485***	0.779***	− 1.719*			
Inn16 ← F_innova	1.775***	1.478***	− 0.575			
Inn17 ← F_innova	1.387***	0.989***	− 0.999			

***p-value < 0.01

**p-value < 0.05

*p-value < 0.10

## Discussion

The present research yielded noteworthy facts about how faculty characteristics relate to the three aspects of their intrapreneurial orientation, i.e., innovativeness, proactivity, and risk-taking. First of all, our results confirm a significant relationship between gender and intrapreneurial orientation of faculty in Kuwaiti HEI. However, we obtained mixed results regarding males versus females’ distinctiveness in proactivity (superior for female faculty), risk taking, and innovativeness (both are superior for male faculty). Those results somehow align with the findings of Serinkan et. al. (2013), which show that females possess more intrapreneurial features than male workers in the Turkish banking sector. Our conclusions also corroborate Yorulmaz et. al. (2016), who also demonstrated that personal innovativeness is higher among female pre-service teachers in Turkey than male peers. Our results contradict, to some extent, Adachi and Hisada’s (2016) study, which posits that, in general, women have less inclination towards intrapreneurship than men. Relatedly, Justus (2021) concluded that female students scored less than their male counterparts in four out of five components of entrepreneurial competence included in her study (i.e., entrepreneurial knowledge, domain-specific interest in entrepreneurship, interest in leadership roles, and entrepreneurial intention). However, they are more likely than men to become intrapreneurs when they work for a small firm. As mentioned earlier, the findings of the study emphasized an increased risk aversion of women in the workplace. This confirms the results of Turro et. al. (2020) in which the perceived consequences of failure refrain female employees from increasing their intrapreneurial engagement.

In light of the results pertaining to educational qualifications, it is clear that Ph.D. holders, in general, have a stronger propensity to intrapreneurship than Master’s holders, at least with regard to the dimensions of proactivity and innovativeness. The only exception concerns faculty engagement towards identifying long-term opportunities and threats for the HEI in which master degree holders tend to be more proactive than Ph.D. holders. This confirms the results of Paray and Kumar (2020) that point out to a higher entrepreneurial intentions among postgraduate students in Indian HEI in comparison to undergraduate students. Casson (1995) and Martiarena (2013) assert that, in general, intrapreneurial employees have higher educational qualifications than the rest. However, our research findings on the impact of education on intrapreneurship in academia are ostensibly not supported in other studies (Adachi & Hisada, 2016; Muange & Kiptoo, 2020; Turro et al., 2013).

Regarding the participant’s specialization, our findings reveal minor dissimilarities between business and engineering faculty regarding their intrapreneurial orientation in favor of participation from business schools. Our evidence here is somehow inconsistent with Paray and Kumar (2020) who demonstrated that management and entrepreneurship students have stronger entrepreneurial attitude than their counterparts in science, engineering and technology. Similarly, the study of Breznitz and Zhang (2020) show a positive relationship between having a non- “science, technology, engineering, and math” degree and graduates’ entrepreneurial activity.

As for teaching experience, our results suggest that this factor is producing stronger proactivity among academics. In addition, participants who accumulated a significant teaching experience are significantly more active in looking for novelties and convincing others to support their ideas. Our findings are relatively aligned with mainstream research emphasizing the positive association between career experience and employee’s predisposition to pursue entrepreneurial opportunities (Bignotti and le Roux, 2020; Shane, 2003). Khayati and Ariail (2020) found that teaching experience is what American and Tunisian university students value the most as faculty attribute. However, other studies pinpointed that propensity towards intrapreneurship is stronger among younger employees than older employees (Adachi & Hisada, 2016).

Another striking finding from the present research shows that faculty with fewer scientific publications adopt a risk-taking posture and aim for higher outcomes even when they face considerable challenges. The study of Khayati and Ariail (2020) shows that the faculty research record is the least attractive for students in their search for quality education. Nonetheless, our results are opposed to those of Rubin and Callaghan (2019) who found a positive association between faculty research productivity and risk-taking propensity. This surprising effect could be attributed to the fact that several HEI in Kuwait is candidates for obtaining renowned international accreditations, whether in business or engineering. Several Kuwaiti HEI are already accredited (e.g., three business schools obtained the accreditation from The Association to Advance Collegiate Schools of Business (AACSB, 2021). The successful completion of the accreditation requirements for universities depends largely on the quality of their academic staff's scientific outcomes. Therefore, we expect that heightened accreditation standards would encourage faculty with lower research records to commit more efforts to compensate for the scientific shortage they have and secure their job positions. Our results are consistent with Roach (2017), who demonstrated that science and engineering doctoral students working in university research labs are more inclined to embrace entrepreneurship and disclose inventions.

Furthermore, this study's outcomes indicate that only faculty with high industry experience are proactive in identifying long-term opportunities and threats for their institutions. In this sense, Hayter (2013) demonstrated that academics with significant business consulting experience are more likely to transform their knowledge into profitable product markets. Other research evidence also supports this assertion as they established that previous intrapreneurial experience produces stronger intrapreneurial behaviors among employees (Bignotti and le Roux, 2020; Turro et al., 2013).

Our findings suggest that earning a significant number of professional certificates is associated with more faculty innovativeness. Also, faculty with extra-academic qualifications are significantly more proactive in promoting new ideas within their working circles. Turro et. al. (2013) demonstrated that cumulating specific training is conducive to increased employee intrapreneurship in common with our research claims. Furthermore, faculty professional development is discussed as an antecedent to organizational change (Bond & Blevins, 2020). In this sense, providing training for staff members involved in explorative activities is likely to improve their ability to identify their organizations' opportunities (Kraus et al., 2019).

Finally, the accreditation status of the latest attended institution does not shape much of faculty members' intrapreneurial propensity. In contrast to general expectations, faculty who were awarded their latest degree from a non-accredited school tend to have stronger perceptions about their capacity to achieve new ideas at work. This finding is likely to dissipate a general taken-for-granted principle in Middle Eastern academic environments hypothesizing that handpicking graduates of reputable Western universities would warrant successful institutions’ successful outcomes. Several universities—particularly in the Gulf region—systematically decline any job applications submitted by candidates holding degrees from non-accredited Western academic institutions. Seemingly, college accreditation does not warrant the faculty’s potential to drive positive change in the HEI. This is potentially due to accreditation process imperfections. For instance, Hussain et. al. (2021) argue that the quality of data measuring students’ outcomes in ABET accreditation of engineering programs is distorted due to several factors including the outdated assessment tools used and the unavailability of digital access.

## Conclusion

Although entrepreneurial orientation has been studied on an organizational or corporate level, it is also applicable to individuals and employees. Intrapreneurship, behaving like an entrepreneur while working within a large organization, reflects entrepreneurial dimensions such as proactiveness, risk-taking, innovativeness, and autonomy. They are essential to universities and academic entities as they are focal for entrepreneurial orientation. The increasingly competitive environment, particularly post the COVID-19 pandemic, pushes organizations, including educational entities, to continue developing sustainable competitive advantage to maintain growth and robust presence in the market. These organizations find core strength in their human capital to innovate and capture opportunities to increase their competitive edge and maximize their value proposition. In this sense, our study demonstrated differentials in how faculty-level characteristics interact with the three dimensions of entrepreneurial orientation in an academic setting. The results highlighted above could be insightful for decision-makers in Kuwaiti educational institutions as they draw attention to insightful facts. For example, graduating from well-established internationally accredited schools does not guarantee stronger entrepreneurial engagement from faculty. On the contrary, industry experience and academic qualifications are strong determinants of faculty proactivity. Regarding gender, while male faculty are risk-takers, females actively identify long-term opportunities and threats for their institutions.

In terms of theoretical implications, this study emphasizes the significance of entrepreneurial orientation in academic settings and adds to the paucity of evidence in that sense. This research is one of the first to empirically test the construct of entrepreneurial orientation with its three underlying dimensions on academic staff. The present study offers also practical implications for HEI as the findings help policy-makers in relating faculty dimensions with specific entrepreneurial outcomes. As a result, the right people will be identified more appropriately for specific positions depending on the degree of innovativeness, proactivity or risk-taking required. For instance, this study refuted a strongly held belief in Gulf states saying that graduates from accredited schools have superior capacity than other to be entrepreneurially engaged. In parallel, it suggested that HEI undertake efforts in training and counseling for those less likely to display strong entrepreneurial dimensions according to our findings.

Future studies can concentrate on the role of online networking in enhancing individual intrapreneurship in HEI. The use of online networking platforms such as LinkedIn and Research Gate and other traditional social media tools enabled faculty to exchange experiences and knowledge with their counterparts worldwide. In this sense, no research has focused so far on the effects that online social interactivity may exert on faculty intrapreneurship.

## Data Availability

Data sources might be available on request.

## Abbreviations

HEI: Higher education institutions
SEM: Structural Equation Modeling
FEO: Faculty entrepreneurial orientation
